# Prediction of Disease Genes Based on Stage-Specific Gene Regulatory Networks in Breast Cancer

**DOI:** 10.3389/fgene.2021.717557

**Published:** 2021-07-15

**Authors:** Linzhuo Fan, Jinhong Hou, Guimin Qin

**Affiliations:** School of Computer Science and Technology, Xidian University, Xi’an, China

**Keywords:** breast cancer, DNA methylation, differentially expressed genes, stage-specific gene regulatory networks, WGCNA

## Abstract

Breast cancer is one of the most common malignant tumors in women, which seriously endangers women’s health. Great advances have been made over the last decades, however, most studies predict driver genes of breast cancer using biological experiments and/or computational methods, regardless of stage information. In this study, we propose a computational framework to predict the disease genes of breast cancer based on stage-specific gene regulatory networks. Firstly, we screen out differentially expressed genes and hypomethylated/hypermethylated genes by comparing tumor samples with corresponding normal samples. Secondly, we construct three stage-specific gene regulatory networks by integrating RNA-seq profiles and TF-target pairs, and apply WGCNA to detect modules from these networks. Subsequently, we perform network topological analysis and gene set enrichment analysis. Finally, the key genes of specific modules for each stage are screened as candidate disease genes. We obtain seven stage-specific modules, and identify 20, 12, and 22 key genes for three stages, respectively. Furthermore, 55%, 83%, and 64% of the genes are associated with breast cancer, for example *E2F2*, *E2F8*, *TPX2*, *BUB1*, and *CKAP2L*. So it may be of great importance for further verification by cancer experts.

## Introduction

Breast cancer is one of the most common malignant tumors in women, and it is the main disease factor that causes cancer deaths in women worldwide. According to statistics (Siegel et al., 2021), breast cancer accounts for 30% of female cancers. In China, breast cancer incidence has two peaks: one is 45–55 years old, and the other is 70–74 years old. From the perspective of age distribution, the incidence of breast cancer gradually increases from the age of 30, and reaches a peak at the age of 55. About 40% of female patients are under 50 (Wild et al., 2020). The symptoms of early breast cancer are unobvious and easy to be overlooked. In the late, cancer cells would metastasize far away, which causes multiple organ diseases, which seriously threatens the lives of patients. However, the current disease genes for breast cancer diagnosis and treatment are far from enough, and it is particularly important to find new candidate disease genes.

Epigenetics is currently a promising field in cancer research. As an important part of epigenetics, DNA methylation has received increasing attention, which is the process of adding methyl groups to DNA molecules and essential for cell development. The functional epigenetic module (FEM) algorithm (Jiao et al., 2014) has verified the inverse correlation between DNA methylation and gene expression, and a large number of researchers have studied the effect of DNA methylation on breast cancer. Bediaga et al. (2010) analyzed the DNA methylation of cancer-related gene regulatory regions in breast cancer paired samples, and effectively identified 15 individual CpG loci that were differentially methylated in breast cancer tumor subtypes, which provides evidence that DNA methylation profile can predict breast cancer subtypes. Based on DNA methylation in whole blood and specific genes, Tang et al. (2016) studied the level of DNA methylation in the blood of breast cancer patients and healthy controls, and found that epigenome-wide blood DNA of breast cancer patients is hypomethylated, and the frequency of *BRCA1* and *RASSF1A* methylation is higher. Lu et al. (2017) explored the relationship between *RUNX3* gene methylation and breast cancer, and the results showed that the hypermethylation of *RUNX3* plays a significant role in the pathological stage and prognosis of breast cancer, which has great potential as a molecular marker for early diagnosis of breast cancer. De Almeida et al. (2019) analyzed the correlation between genome-wide methylation and gene expression by matching breast cancer DNA methylation with normal tissues in the TCGA, and identified new DNA methylation markers, including *PRAC2*, *TDRD10*, *TMEM132C*, etc., are expected to become diagnostic and prognostic markers of breast cancer.

There are also bioinformatics experts who study breast cancer based on biological molecular networks. Cai et al. (2019) used WCGNA to screen out the gene modules related to the risk of breast cancer metastasis, combined with the PPI network to screen out five key genes related to breast cancer progression and verified them. Lin et al. (2020) constructed a PPI network to screen hub genes, used modular analysis and survival analysis to identify potential target genes and pathways that may affect the occurrence and development of HER-2 positive breast cancer. Tang J. N. et al. (2018) identified five candidate biomarkers by analyzing the co-expression network, and used candidates in the basic and clinical research of breast cancer. Xi et al. (2018a) detected that *TP53* and *PNRM1* driver genes play an important role in breast cancer through matrix tri-factorization framework with pairwise similarity constraints. Guo et al. (2017) explained the mechanism of breast cancer development by identifying key pathways in breast cancer tissue and constructing the network of transcription factors (TFs) and microRNA (miRNA). Qiu et al. (2019) established the gene co-expression network for identifying modules related to breast cancer development, and discovered hub genes that may be used as markers of invasive breast cancer. Xi et al. (2018b) discovered mutated driver genes by using a robust and sparse co-regularized matrix factorization framework with prior information from mRNA expression patterns and interaction network. By combining the subspace learning framework, Xi et al. (2020) proposed the DriverSub algorithm to infer specific driver genes from heterogeneous breast cancer samples.

In this article, we propose a computational framework to predict candidate stage-specific disease genes of breast cancer based on the stage-specific gene regulatory networks. Firstly, we screen out differentially expressed genes and hypermethylated/hypomethylated genes by comparing tumor samples and normal samples. Secondly, we construct and analyze three stage-specific gene regulatory networks by taking stage information into account. Thirdly, we identify stage-specific modules by module division. Finally, we predict candidate stage-specific disease genes.

Our contributions consist of two points:

(1) We integrate stage information and DNA methylation information to construct a stage-specific gene regulatory network for breast cancer, which may help doctors identify patient’s disease stage more quickly and design better treatment strategy.
(2) The proposed computational framework is effective in predicting breast cancer related genes, which will help experts to explore the molecular mechanisms of breast cancer.

## Materials and Methods

**FIGURE 1:**
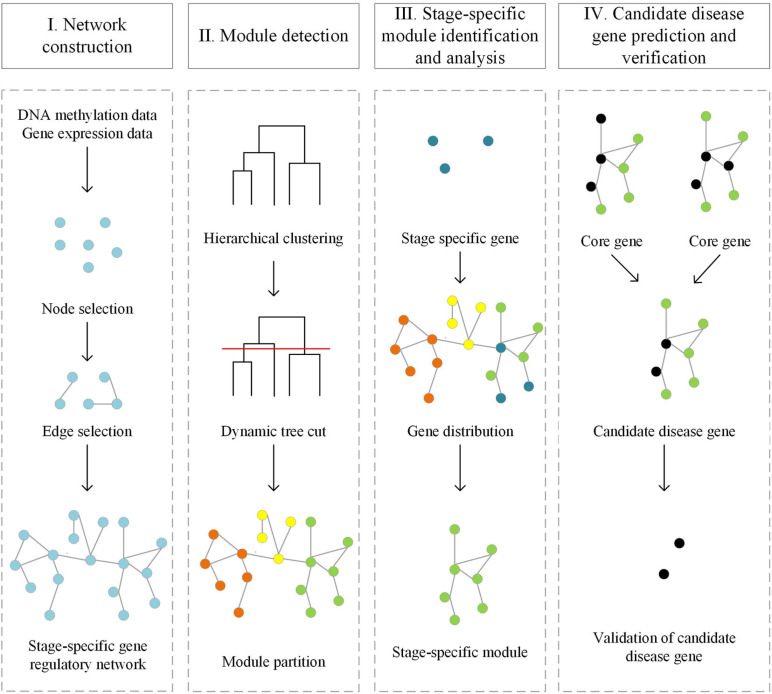
Workflow of the computational framework for predicting disease genes based on stage-specific gene regulatory network.

Our computational framework for predicting candidate disease genes includes four parts: Stage-specific gene regulatory networks construction, Module division, Topological properties analysis and gene set enrichment analysis, Candidate disease genes prediction (Figure 1).

### Data Preprocessing

We download breast cancer phenotype data, gene expression profile and DNA methylation data from TCGA (Tomczak et al., 2015) (The Cancer Genome Atlas), which is currently the largest public cancer database, containing nearly 40 common cancer types and tens of thousands of samples. There are 60,484 genes and 1,217 samples in the gene expression profile, and 485,578 CpG sites and 890 samples in the DNA methylation data, respectively. We only retain the sample pairs, i.e., each tumor sample has a corresponding normal sample. Then, we divide the samples according to the stage information, and obtain 29 pairs, 94 pairs, 32 pairs of samples in stage I, stage II, and stage III, respectively. There are only two pairs of samples in stage IV that meet the experimental standards, which is not convincing. Therefore, we exclude samples in stage IV. For the DNA methylation data, we first convert the CpG site into the gene. As there are many CpG sites in a gene, we just use their mean β value to represent the DNA methylation level of the gene. For the gene expression profile, we download normalized FPKM data and filter out 15% genes with missing values. Then we select samples that have both cancer tissue and normal tissue.

The Gene Expression Omnibus (GEO) (Barrett et al., 2005) database includes a large amount of sequencing data and omics data, which is comprehensive and free. We download the GSE15852 and GSE69914 datasets from GEO (Liu et al., 2017). GSE15852 is the raw gene expression data from 43 human breast cancers and their corresponding normal tissues. GSE69914 is DNA methylation profiling of 50 normal samples from healthy women, 42 matched normal-adjacent breast cancer pairs (84 samples), 263 unmatched breast cancers, seven normal samples from BRCA1 carriers and four BRCA1 breast cancers. We only use 42 matched pairs of normal-adjacent breast cancer.

### Differentially Expressed Genes and Hypomethylated/Hypermethylated Genes Identification

**FIGURE 2:**
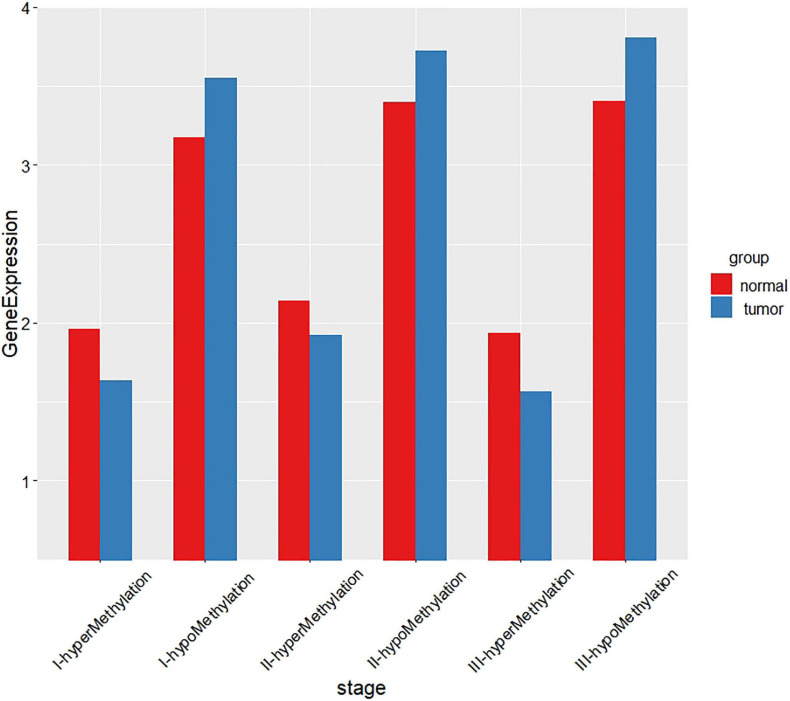
The relationship between DNA methylation and gene expression of each stage.

For the gene expression profile, we use Limma (Ritchie et al., 2015) in the R package to screen the differentially expressed genes, and use *p*-value less than 0.05 and |log FC| less than 0.5 as the threshold. For the DNA methylation data, we define β value greater than 0.8 as hypermethylated genes and β value less than 0.2 as hypomethylated genes. Then we take the intersection of the differentially expressed genes and the hypermethylated/hypomethylated genes and obtain 1,027 genes, 1,012 genes, and 1,220 genes in stage I, stage II, and stage III, respectively. Then we compare the relationship between the DNA methylation profile and gene expression profile, and find that the higher the gene methylation level, the lower the gene expression. And the results are shown in Figure 2.

### Stage-Specific Gene Regulatory Networks Construction

Gene Regulatory Network database (GRNdb) (Fang et al., 2020) is a gene regulatory network database, which includes a large number of human and mouse transcription factor and target gene pairs. We download the TF-target gene pairs from the GRNdb, and filter out the pairs in which the target genes are differentially expressed genes and hypermethylated/hypomethylated genes (Qin et al., 2019). Then we calculate the Pearson Correlation Coefficient (PCC) for each TF-target gene pair based on their expression level, and the cut-off is set as 0.5 and construct stage-specific gene regulatory networks.

### Module Division

We use WGCNA (Langfelder and Horvath, 2008) to divide the stage-specific gene regulatory network into modules. Firstly, we perform hierarchical clustering on the three stage-specific gene regulatory networks to generate a hierarchical clustering tree. Then, we use the Dynamic Tree Cut algorithm (Langfelder et al., 2008) to divide the above-generated hierarchical clustering tree and ensure that the number of molecules in each module is at least 30.

### Topological Properties Analysis and Gene Set Enrichment Analysis

Hub genes are important for biological processes. We identify and compare hub genes for each gene regulatory network. We perform topological analysis of stage-specific gene regulatory networks using Cytoscape (Shannon et al., 2003), including degree distribution, centrality distribution, and so on. Then, we perform gene set enrichment analysis using Metascape (Zhou et al., 2019).

### Candidate Disease Gene Prediction

We filter out candidate disease genes from the above modules and network topological information. Then, we checked them by known disease-related genes from OMIM, COSMIC, and DAVID. Online Mendelian Inheritance in Man (OMIM) (Hamosh et al., 2005) mainly covers the relationship of genes and diseases, the relationship of genes and phenotypes, and some clinical features. Catalog of Somatic Mutations in Cancer (COSMIC) (Sondka et al., 2018) integrates cancer somatic mutations and provides cancer gene mutation map data information. DAVID (Huang et al., 2009) integrates biological data and analysis tools and provides systematic and comprehensive biological function annotation information for large-scale gene or protein lists. Furthermore, we check the association of the rest of the candidate disease genes and breast cancer in PubMed (Shashikiran, 2016).

## Results

### Stage-Specific Gene Regulatory Network Construction

We filter out the TF-target gene pairs whose target genes are not differentially expressed genes and hypermethylated/hypomethylated genes, and use the PCC cut-off 0.5 to construct stage-specific gene regulatory networks. There are 1,129, 1,066, and 1,339 nodes and 4,429, 4,879, and 6,461 edges, respectively.

### Module Division

**FIGURE 3:**
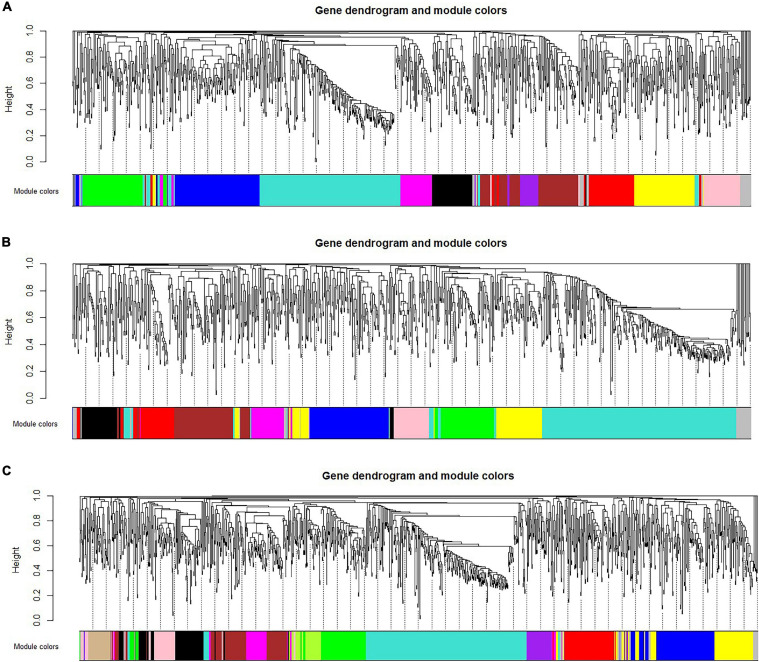
Module division results of each stage. **(A)** Stage I, **(B)** Stage II, **(C)** Stage III.

We use WGCNA to divide three gene regulatory networks into modules and the results are shown in Figure 3. We find that the first-stage network is divided into 11 modules, of which the turquoise module contains up to 270 genes. The number of genes in the remaining modules ranges from 40 to 149. The second-stage network is divided into 10 modules, of which the turquoise module contains 337 genes. The number of genes in the remaining modules ranges from 40 to 125. The third-stage network is divided into 13 modules, of which the turquoise module contains 337 genes. The number of genes in the remaining modules ranges from 30 to 142. In particular, the gray modules contain genes that are not classified into any module and discarded. The detailed information of the number of genes in each module is shown in Table 1.

**TABLE 1:** Gene distribution of each module.

**Module**	**Gene count**	**Specific gene count**	**Module**	**Gene count**	**Specific gene count**	**Module**	**Gene count**	**Specific gene count**
S1_black	71	5	S2_black	66	4	S3_black	99	11
S1_blue	149	11	S2_blue	125	5	S3_blue	142	8
S1_brown	120	28	S2_brown	116	3	S3_brown	120	26
S1_green	106	5	S2_green	90	9	S3_green	110	25
S1_grey	42	4	S2_grey	40	3	S3_greenyellow	51	14
S1_magenta	63	4	S2_magenta	58	3	S3_grey	30	2
S1_pink	66	10	S2_pink	58	6	S3_magenta	61	4
S1_purple	40	2	S2_red	68	3	S3_pink	70	14
S1_red	95	8	S2_turquoise	337	21	S3_purple	54	10
S1_turquoise	270	13	S2_yellow	108	2	S3_red	104	15
S1_yellow	107	1				S3_tan	45	2
						S3_turquoise	337	47
						S3_yellow	116	8

*S1, S2, and S3 represent stage I, stage II, and stage III, respectively.*

We identify differentially expressed genes that only exist in one stage as the stage-specific genes and obtain 92 genes, 60 genes, and 187 genes in stage I, stage II, and stage III, respectively. Then we count the distribution of these genes in each module, as shown in Table 1. We find that the specific genes in stage I are mainly distributed in the S1_brown module, S1_turquoise module and S1_blue module, the specific genes in stage II are mainly distributed in the S2_turquoise module, and the specific genes in stage III are mainly distributed in the S3_turquoise module, S3_brown module and S3_green module. Therefore, we regard these seven modules as the specific modules of corresponding stage.

### Topological Properties Analysis and Gene Set Enrichment Analysis

We perform network topological analysis for seven specific modules using Cytoscape. For the degree distribution, the degrees of S1_turquoise module, S2_turquoise module, and S3_turquoise module are mainly distributed between 100 and 400, and the degrees of S1_brown module, S1_blue module, S3_brown module, and S3_green module are mainly distributed between 50 and 100, respectively. And the degree distribution of each module conforms to the power law distribution. The betweenness centrality of most nodes in each module is at a high level. The closeness centrality of most nodes in each module ranges from 0.5 to 0.9. These values indicate that the network corresponding to each module is a dense graph, so the hub genes screened by these three parameters are all core genes.

**FIGURE 4:**
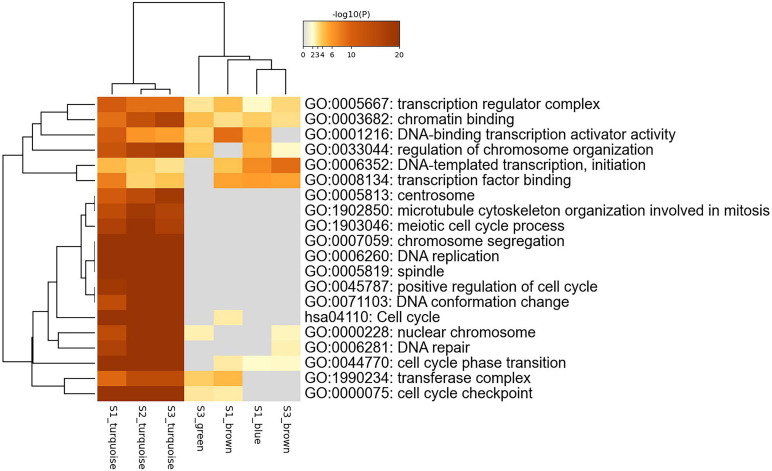
Joint enrichment analysis of seven specific modules.

We use Metascape to perform the joint enrichment analysis on the genes in the seven specific modules, and set *p*-value cut-off 0.01. The joint enrichment results are shown in Figure 4. The most significant enrichment item for each module is shown in Table 2. According to Figure 4 and Table 2, S1_turquoise, S2_turquoise, and S3_turquoise modules are roughly identical, and these significant pathways are all related to cell transcription and cycle regulation. S3_green, S1_brown, S1_blue, and S3_brown modules are closely related to each other, and these significant pathways are mainly related to gene transcription. In addition, transcription regulation complex (GO:0005667) and chromatin binding (GO:0003682) are the common enrichment items of the seven specific modules. The results show that the stage-specific modules have strong functionality and the genes within the modules are highly correlated.

**TABLE 2:** Functional enrichment analysis.

**Module**	**Term**	**Description**	**Log10(P)**	**Count**
S1_turquoise	GO:0044770	Cell cycle phase transition	−32.060	52
	GO:0051301	Cell division	−31.343	50
	GO:0006260	DNA replication	−21.835	30
S1_blue	GO:0022411	Cell component disassembly	−8.88	18
	GO:0001046	The core promoter sequence specifically binds to DNA	−8.71	7
	GO:0070897	Transcription pre-priming complex assembly	−3.78	6
S1_brown	GO:0001228	DNA binding transcription activator activity	−9.13	15
	GO:0001227	DNA binding transcription repressor activity	−6.08	10
	GO:0004879	Nuclear receptor activity	−5.54	5
S2_turquoise	GO:0044770	Cell cycle transition	−41.180	66
	GO:0007059	Chromosome segregation	−38.573	50
	GO:0005819	Spindle	−27.162	42
S3_turquoise	GO:0044770	Cell cycle transition	−44.41	69
	GO:0098687	Chromosome region	−38.25	50
	hsa04110	Cell cycle	−28.49	29
S3_brown	GO:0006352	DNA template transcription	−9.100	12
	GO:0001046	The core promoter sequence specifically binds to DNA	−7.678	6
	GO:0034655	Catabolism of nucleobase-containing compounds	−6.932	14
S3_green	GO:0016570	Histone modification	−6.678	12
	GO:0005697	Telomerase holoenzyme complex	−5.816	4
	GO:0034243	Macromolecule methylation	−5.194	5

### Candidate Disease Gene Prediction

We predict disease genes based on correlation matrix and network topological properties. Firstly, we calculate the correlation matrix of genes at each specific module, and select genes with correlation cut-off 0.8 and *p*-value cut-off 0.05 as the core genes of each module. Then, we sort the degree distribution, betweenness centrality and closeness centrality of each gene in the seven modules, and select the top 5% as the core gene of each module. The intersection of core genes selected by these two methods are considered as candidate disease genes.

We obtain 20 candidate disease genes in stage I, such as *E2F2*, *E2F8*, *TPX2*, etc., 12 genes in stage II, such as *KPNA2*, *CKAP2L*, *CBX3*, etc., and 22 genes in stage III, such as *RAD21*, *FBXO5*, *CCNE2*, etc. A complete gene list of each stage is shown in Table 3. *E2F2, CKAP2L* and *CBX3* are genes shared by three stages. For the remaining candidate genes at different stages, we compare their gene expression data and find that they are indeed different at different stages. And the results are shown in the Supplementary Figures 1∼2.

**TABLE 3:** Candidate disease genes at each stage.

**Stage**	**Candidate disease genes**
Stage I	E2F2*, E2F8^#^, TPX2*, BUB1*, CKAP2L^#^, CBX3^#^, CASC5^#^, KPNA2^#^, LMNB1, NEK2*, TTK*, SLC25A36, CREBRF, ZC3H6, PAN2, BTAF1, SLC25A39, DDX49, SLC39A1^#^, MRPS12
Stage II	E2F2*, E2F8^#^, TPX2*, KPNA2^#^, CKAP2L^#^, CBX3^#^, DDIAS, BUB1*, CCNE2^#^, CASC5^#^, SPDL1, TOP2A*
Stage III	E2F2*, RAD21^#^, FBXO5^#^, CCNE2^#^, CBX3^#^, STIL^#^, CKAP2L^#^, PCNA*, NEK2*, TTK*, CSE1L^#^, H2AFZ^#^, NR2F6, TRAPPC6A, IGSF8, FDXR, SLC39A1^#^, EXOSC5, RBBP5, KDM5B^#^, H3F3A, CDC42SE1
Common genes	E2F2, CKAP2L, CBX3

*Genes verified by OMIM, COSMIC, DAVID. #Genes verified by PubMed.

### Candidate Disease Gene Verification

In order to determine whether the selected candidate disease genes are effective in the diagnosis and treatment of breast cancer, we use OMIM, COSMIC, and DAVID to verify the candidate genes, and obtain seven genes related to breast cancer. *BUB1* is mitotic checkpoint serine, *E2F2* is a transcription activator, *NEK2* is a serine/threonine-protein kinase, *TPX2* is the target protein for *Xklp2*, *TTK* is essential for spindle establishment and centrosome replication, *PCNA* is the proliferating cell nuclear antigen, and *TOP2A* is DNA topoisomerase 2-alpha. Most of these genes are related to cell proliferation and transcription.

We search the rest candidate disease genes related to the genes in PubMed, and verify whether the genes are related to breast cancer. Kos et al. (2020) found *STIL* is an important prognostic and predictive biomarker for triple-negative breast cancer and HER2-positive breast cancer. At present, there have been studies on pathological assessment of breast cancer based on *STIL*, which is a key step for molecular markers to move toward clinical treatment. Based on the study of differentially expressed hub genes, Qi et al. (2019) proposed that the overexpression of *CCNE2, H2AFZ, TOP2A* is closely related to the diagnosis and poor prognosis of breast cancer. Yuksel et al. (2015) found the overexpression of *CSE1L* has a certain relationship with the distant metastasis of breast cancer and may be a valuable prognostic tool. Tang J. et al. (2018) used WGCNA to construct a co-expression network and found *FBXO5* and *TPX2* are related to the poor prognosis of breast cancer. Liang et al. (2017) found *CBX* family proteins have epigenetic regulatory functions, among which the high expression of *CBX3* is related to the worsening of recurrence-free survival rate of breast cancer patients. Liu et al. (2018) found *E2Fs* are transcription factors that affect cell proliferation, differentiation and apoptosis, and the high expression of *E2F8* is also related to the deterioration of patients’ recurrence-free survival rate, and can be used as a potential target for individualized treatment of breast cancer patients. Zhang et al. (2019) showed that *KDM5B* is up-regulated in breast cancer and many other cancers and its expression is positively correlated with breast cancer metastasis. Duan et al. (2020) and Liu et al. (2020) showed the expression of *KPNA2* and *SLC39A1* in breast cancer tissues is significantly up-regulated, which can regulate the development of breast cancer and provide new targets for breast cancer treatment. *NEK2* is a kind of serine, which plays an important role in mitosis. Cappello et al. (2014) and Chen et al. (2020) have proven *NEK2* is a target for breast cancer. Atienza et al. (2005) has shown through experiments that *RAD21* can enhance the anti-tumor activity of chemotherapeutics by inducing DNA damage and is a new target for cancer drugs. Based on survival analysis and mutation analysis, Fu et al. (2019) found that the high expression of *CKAP2L* and *CASC5* is closely related to the poor prognosis of breast cancer patients. These verified genes are shown in Table 3.

In summary, we detect 20, 12, and 22 candidate disease genes for three stages, respectively. Through PubMed search, 11, 10, and 14 genes are verified, respectively. That is 55%, 83%, and 64% of the candidate disease genes are proved to be related to the diagnosis and treatment of breast cancer, respectively, such as *E2F2*, *E2F8*, *TPX2*, *BUB1*, *CKAP2L*, etc. The results show the effectiveness of our computational framework for predicting disease genes.

We also use GSE15852 gene expression profile and GSE69914 DNA methylation profile to verify the validity of the proposed computational framework. Firstly, we screen out 79 differentially expressed genes and hypermethylated/hypomethylated genes. Secondly, we combine with the TF-target gene pairs and construct a gene regulatory network with 195 nodes and 313 edges. Thirdly, we divide the gene regulatory network into four modules: 76 genes in turquoise module, 68 genes in blue module, 18 genes in gray module, and 33 genes in brown module, respectively. In particular, the gray module contains genes that are not classified into any module and discarded. Finally, we screen the candidate disease genes of each module based on correlation matrix and network topological properties, and obtain four genes in turquoise module, four genes in blue module, and two genes in brown module, respectively. In detail, these genes are *H2AFZ, NPM1, MAF, NR3C1, PTGER3, TCF4, IRF1, RARB, CHD2*, and *SMAD4.* Except *PTGER3* and *CHD2*, other genes have been verified. This means that our method is effective, and it may help experts explore breast cancer related genes.

## Discussion

At present, the proposed computational framework has only been tested on breast cancer, and satisfactory results have been obtained. In the future, we will try to apply this framework to other types of diseases for discovering more disease-related genes.

## Conclusion

We propose a computational framework to predict candidate stage-specific disease genes for breast cancer based on stage-specific gene regulatory networks. And we conduct experiments using two breast cancer data sets and find that most predicted genes are related to breast cancer, which shows that our method is effective. We also predict some candidate disease genes that need to be further verified. Nevertheless, our research has some limitations. Our proposed computational framework is based on the public TCGA and GEO datasets, and the noise affects the analysis results. Another limitation is that we should integrate more omics data so that more disease genes may be predicted more accurately.

## Data Availability Statement

The original contributions presented in the study are included in the article/Supplementary Material, further inquiries can be directed to the corresponding author.

## Ethics Statement

Ethical review and approval was not required for the study on human participants in accordance with the Local Legislation and Institutional Requirements. Written informed consent for participation was not required for this study in accordance with the National Legislation and the Institutional Requirements.

## Author Contributions

JH and LF conceived and developed the computational framework for predicting disease genes and wrote the manuscript. GQ provided important feedback in the framework process and edited the manuscript. All authors have made significant contributions to the completion and writing of the manuscript and read and approved the final manuscript.

## Conflict of Interest

The authors declare that the research was conducted in the absence of any commercial or financial relationships that could be construed as a potential conflict of interest.

## Supplementary Material

Supplementary Figures 1∼2 The gene expression boxplot of different candidate disease genes at different stages.
